# Fusion of Multi-Modal Features to Enhance Dense Video Caption

**DOI:** 10.3390/s23125565

**Published:** 2023-06-14

**Authors:** Xuefei Huang, Ka-Hou Chan, Weifan Wu, Hao Sheng, Wei Ke

**Affiliations:** 1 Faculty of Applied Sciences, Macao Polytechnic University, Macau 999078, China; xuefei.huang@mpu.edu.mo (X.H.); chankahou@mpu.edu.mo (K.-H.C.); weifan.wu@mpu.edu.mo (W.W.); shenghao@buaa.edu.cn (H.S.); 2 Engineering Research Centre of Applied Technology on Machine Translation and Artificial Intelligence of Ministry of Education, Macao Polytechnic University, Macau 999078, China; 3 State Key Laboratory of Virtual Reality Technology and Systems, School of Computer Science and Engineering, Beihang University, Beijing 100191, China; 4 Beihang Hangzhou Innovation Institute Yuhang, Yuhang District, Hangzhou 310023, China

**Keywords:** dense video caption, video captioning, multi-modal feature fusion, feature extraction, neural network

## Abstract

Dense video caption is a task that aims to help computers analyze the content of a video by generating abstract captions for a sequence of video frames. However, most of the existing methods only use visual features in the video and ignore the audio features that are also essential for understanding the video. In this paper, we propose a fusion model that combines the Transformer framework to integrate both visual and audio features in the video for captioning. We use multi-head attention to deal with the variations in sequence lengths between the models involved in our approach. We also introduce a Common Pool to store the generated features and align them with the time steps, thus filtering the information and eliminating redundancy based on the confidence scores. Moreover, we use LSTM as a decoder to generate the description sentences, which reduces the memory size of the entire network. Experiments show that our method is competitive on the ActivityNet Captions dataset.

## 1. Introduction

A dense video caption is an abstract representation of the important events in unedited videos that may contain different scenes. Thanks to the popularity of Internet resources and mobile devices, the amount of video data is increasing, and the types of which are becoming very rich. As an important way to disseminate information, videos have become inseparable from people’s daily lives, and the use of live videos has become a trend. The tasks of dense video captions are to effectively label different types of content, and can be applied to various scenarios in videos towards extraction of higher-level semantics. For instance, monitoring traffic safety, finding target people, assisting in reviewing video content, and improving network security [1,2,3]. In contrast to tasks such as object recognition and tracking, video captioning requires a combination of computer vision [4,5,6,7] and Natural Language Processing (NLP) techniques [8]. In addition to spatial and temporal information, there is also the contextual information contained in the video, including sound effects and speeches. It makes dense captioning much more challenging. Video captioning requires the computer to not only unambiguously recognize objects in the video, but also to understand the relationships between the objects. Finally, it requires the computer to express the contents of the video in logical terms of human languages [9,10,11].

The emergence of deep learning [12] first achieved breakthroughs in image caption tasks, and the encoder–decoder framework was quickly transferred to video caption domains by researchers who have obtained various results [13,14]. Video caption tasks aim to generate a natural language sentence that summarizes the main content of a given short video. However, most videos do not contain only one event, but are composed of multiple scenarios. The events in a given video are usually related to each other, and most events are action-oriented and can even overlap [15,16]. These characteristics make it difficult for a single sentence to fully express the complex content of the video. To solve this problem, a dense video caption task was proposed to generate a natural language paragraph that describes all of the important events and details in a given video. The dense video caption task accurately expresses the complex content of the video through multiple compound sentences, which is more in line with human needs for artificial intelligence [17].

**Figure 1:**
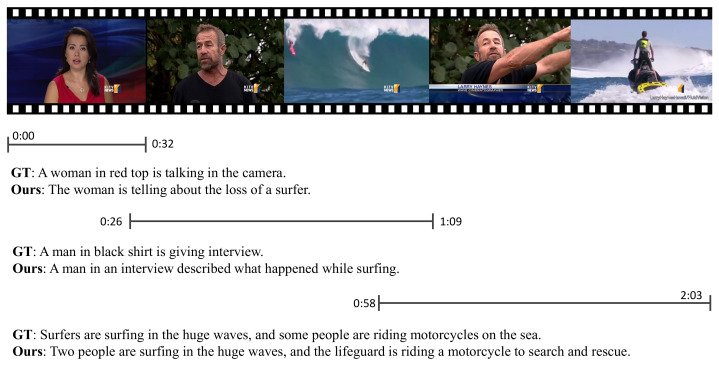
Example video with the predictions of our model alongside the ground truth.

Figure 1 shows one of the successive frames of an unedited video containing different scenes. It can be seen that this video converts four different scenes, and it is difficult to fully capture all of the content of the video without the help of a dense video caption task. As a result, the generated text is incomplete and incoherent. Furthermore, Figure 1 indicates the importance of combining audio patterns to generate text descriptions. As we can see, the first segment of the video determines the caption as “a woman in a red top talking to the camera” from a purely visual perspective. In fact, the woman in this video is a broadcaster providing the news via audio, and talking about a surfer who accidentally got lost while attempting a huge wave. From the above example, we can conclude that the audio enables our model to produce captions that match the full content of the video more closely. Therefore, it is important to integrate audio features into dense video caption tasks, which help computers to comprehend relatively abstract videos and express rich scenes in text.

Building on previous work, we focus on how to fully integrate features into video and audio sequences as vectors of different lengths. The main contributions of this paper are as follows:

(1) We introduce a new framework for dense video caption generation. Such framework makes use of the Transformer’s multi-head attention module to efficiently fuse video and audio features in video sequences, thus improving the accuracy and richness of the model-generated captions.
(2) We propose a confidence module to select major events, which addresses the problem of unequal recall and precision after using fused video–audio features, making the fused audiovisual features more effective in generating descriptive texts.
(3) We employ LSTM as a decoder for sentence representation, which has the advantage of long-term memory to meet the requirements of text description generation, and also enhances the overall computational efficiency of the framework.
(4) We show that our framework is competitive with existing methods on the ActivityNet Captions dataset.

We arrange the following content as below. Section 2 gives an overview of the related work in video captioning and the deep learning approaches. Section 3 describes the structure of our multi-modal approach and the technical details. The setup of experiments, the discussions of results, and the comparisons with other methods are presented in Section 4. Finally, we conclude the paper in Section 5.

## 2. Related Work

Video captioning is an introduction to what the video contains in logical sentences. A typical video sequence is formed by playing more than a dozen frames (images) per second quickly. Therefore, the initial methods for dense video captioning were largely inspired by the image caption field, especially the encoder–decoder structure based on deep learning, which can encode the visual features and decode them into natural language sentences [18,19,20]. The sequence-to-sequence–video-to-text [21] (S2VT) model follows this idea, where a certain number of frames are extracted from the video as images. The encoding part uses the VGG [22] network to process the characteristics of the input data, and adds the optical flow method as an auxiliary. Then, the extracted features are averaged, and a text description is generated in the decoding part using LSTM [23]. However, due to the particularity of video, this method does not take into account the timing information contained in the video, and the generated text description is not detailed enough.

The emergence of convolutional three-dimension networks (C3D) has solved the above problems to a certain extent [24]. It adds the time dimension to the original structure of a 2D CNN, which is more conducive to processing complex video data, and the extracted video features are more comprehensive. Therefore, C3D gradually occupies a major position in the field of video captioning [25,26,27,28], and many other projects use it as an encoder for the feature extraction of videos. The inflated 3D convNet (I3D) adds optical flow features on the basis of C3D [29]. The weight of the 2D CNN model pre-trained on ImageNet is used as the initial parameter to train the model, which further improves the performance of video feature extraction. The pseudo-3D residual network (P3D) decomposes 3D convolutions into two-dimensional space convolutions and one-dimensional time convolutions, and adds the concept of residual connection to increase the overall depth of the network, and obtains good results [30].

Dense video caption [31] has raised the video caption task to a new level. On the basis of the S2VT model, the original short description text is extended to the problem of caption generation based on regional sequences, which improves the comprehensiveness and diversity of descriptions and maintains the accuracy. For this task, the ActivityNet Captions dataset is also proposed, which has a high position in the field of dense video captioning [32,33]. Yu et al. proposed the concept of converting a video into an article, using multiple sentences to form a long text paragraph to summarize the video substance [34]. The decoding part of the model is divided into two modules: sentence generation and paragraph composition. The description text as referenced in the dataset is used as a part of the input to train the model to learn the correlation information between sentences. The effective results of this work have inspired the research on video caption tasks to a certain extent. The single-stream temporary action proposal (SST) [35] model obtains the timing information of the video by filtering the threshold, and uses the attention mechanism to analyze the information; the output video features are input to the decoder as the initial state. The Meteor [36] score of this model on the ActivityNet Captions dataset is 9.65, which is higher than that of other models at the same stage.

Because of the outstanding performance, recurrent neural networks (RNN) in the NLP field are applied to video caption tasks. Most models use the characteristics of LSTM that can remember long sequences as a decoder to generate description text. Pan et al. proposed the LSTM-E model [37], which is on the basis of traditional cross-entropy loss. A correlation loss is added to allow the model to learn both semantic relationships and visual content, fully associated sentences used as references with visual features, and improve the accuracy of the output. The boundary-aware encoder [38] model uses LSTM as the encoding part, and proposes a recurrent video coding scheme, which can better explore and use the hierarchical structure in the video, and enhance the matching degree with the timing information in the video.

More importantly, researchers have sought to apply the attention mechanism to the field of video captioning and achieved good results. Yao et al. proposed to introduce attention weight α on the basis of an S2VT model to calculate features of time series, paying high attention to important information in the video, and ignoring some interference or unimportant information [39]. The evaluation index of this behavior is higher than other models in the same period. The spatio-temporal and temporo-spatial attention (STaTS) model [40] takes the language state as the premise, complements the spatial and temporal information of a video through two different attention combinations, and proposes an LSTM-based time sequence function (sorting attention), which can be used to capture actions in the video.

In order to solve the problem that the LSTM structure cannot be trained in parallel, the Transformer frame builds a global relationship on the semantic information of the reference description statements in the dataset based on the attention mechanism, and has achieved good results in dealing with the problem of missing details of video caption tasks [41,42,43,44,45]. Wang et al. proposed a training model based on a Transformer (EEDVC) [46], with one encoder corresponding to two decoders. The video is divided according to different events, and each extracted event is described separately. However, this approach relies too much on the quality of video feature extraction, and the time information captured in the video is insufficient. Wang et al. proposed the parallel decoding method (PDVC) [47] on the basis of EEVDC, which enhanced the model’s learning of video features and semantic relationships in reference sentences through two different parallel Transformer methods. The diverse paragraph captioning for untrimmed videos (TDPC) [48] uses the Transformer framework, and adds a dynamic video memory module to interpret the global features of video in stages, taking into account the accuracy and diversity of text descriptions.

The description text generated only for the visual modal information in the video cannot cover all of its content [49,50]. The enhanced topic-guided system [51] introduces the Mel frequency cepstrum coefficient (MFCC) [52] to extract the audio feature information in the video, and fully integrates the visual and audio features, to achieve the purpose of an all-around description. Iashin et al. continued this theory, using I3D and VGGish [53] to extract features of visual and audio modes in the video, and introduced a bi-Transformer framework to abstract video expression [54]. The multi-modal dense video caption (MDVC) model [55] builds upon the Transformer architecture, where the visual, audio, and speech in the video are used as input data, and finally converted into text descriptions. Chang et al. proposed an EMVC method [56], using visual-audio cues to generate event proposals, and developed an attention gate that dynamically fused and adjusted the multi-modal information control mechanism. Hao et al. proposed three different depth fusion strategies for multi-modal information in videos, trying to maximize the advantages of audio-visual resonance [57]. Park et al. combined the human face information in the video, extracted the spatio-temporal features in the video using I3D, and combined it with the Transformer architecture to predict the relationship between different IDs and objects [58].

Based on the above analysis, we summarize some methods for video captioning in Table 1. In addition, we can clearly understand that the video caption field has achieved significant performance improvement in recent years, but it still faces some challenges and problems. On the one hand, video captioning needs to fully utilize the multimodal data features in videos, such as visual, audio, and text, but most current methods only focus on visual features and ignore the importance of other features for generating accurate and rich descriptions. On the other hand, video captions need to generate natural language descriptions that are highly relevant and grammatically correct for the video content, but most of the current methods only use template-based or sequence learning-based language models, lacking the modeling of complex relationships and logical reasoning abilities between video and text.

Therefore, how to better utilize the multi-modal data features in videos, and how to more effectively model the complex relationships and logical reasoning abilities between video and text, are still challenges that need to be focused on and solved in the future research of this field.

## 3. Methodology

Considering the importance of audio patterns in a video and combining the above research points, a dense video caption generation model that fully integrates visual and audio patterns is proposed. This model applies the I3D and VGGish approaches to extract visual and audio features, respectively. Moreover, the output features produced by these approaches are always presented in different sizes, we thus introduce a multi-head attention module to integrate the extracted visual and audio features. Finally, the LSTM is used as a decoder to implement a descriptive textual representation of the video content.

### 3.1. Model Overview

**Figure 2:**
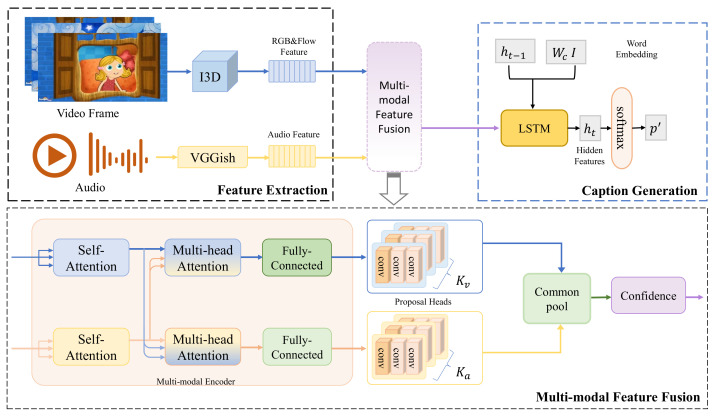
Overall framework of the proposed model.

The framework of the proposed model consists of three parts: feature extraction, multi-modal feature fusion, and caption generation. Figure 2 presents the entire framework and the data flow between the three parts in a schematic diagram.

**Feature Extraction**  Since there are size differences between visual and audio features, they need to be extracted separately to remove noise and redundancy. For the visual pattern features, the I3D network is applied to achieve the extraction of spatial features present in the video, while optical flow features are also added to further improve the performance. Next, VGGish is used to extract a selection of audio features that can effectively convert the audio stream into a feature vector corresponding to natural language elements.
**Multi-Modal Feature Fusion**  The features extracted from visual and audio modalities produce vectors of different dimensions that cannot be directly fused. Therefore, a multi-model attention fusion module is proposed as an encoder based on the Transformer framework, aiming to fully fuse the audio and visual features for information resonance. Furthermore, a confidence module is added to filter the major information in this part.
**Caption Generation**  We employ LSTM to retain the attributes of lengthy sequences as a decoder. The proposals evaluated by the confidence module serve as the initial state input of the decoder, which simulates the distribution in the vocabulary encoded by the embedded position. Finally, a detailed textual description for the video is generated automatically.

### 3.2. Feature Extraction

Currently, there is no method to extract both visual and audio features from a video simultaneously. These features can only be extracted from different modalities. As noted in Section 2, CNN is a highly regarded method in the field of computer vision that outperforms in dense video caption. Since a video is essentially a collection of still images that contain temporal information, relying solely on 2D CNN networks to extract information from video frames ignores the correlation between frames and thus fails to fully extract the rich information in the video. Therefore, we recommend applying the I3D pre-trained on the Kinetics dataset [59] as the backbone for extracting visual features from the video. This approach adds optical flow features to the spatio-temporal features of the video that can be learned by the C3D approach. We also train the RGB and optical flow networks separately as the input features, then average them during testing. Additionally, I3D contains a deeper network structure and a multi-branch structure, allowing for reduced parameters and increased efficiency. In practice, we apply I3D to extract RGB and optical flow features from each video frame, then combine these two features and encode them using linear layers to achieve a simple, compact, and well-suited network model for intensive video annotation tasks.

We also use the VGGish network to extract audio features from videos. Numerous studies have demonstrated that VGGish outperformed traditional methods for audio extraction. This is achieved by combining the deep structure of the VGG network with log-mel features and training on a large amount of audio data from the Audioset dataset [60]. The pre-trained parameters show strong generalization capabilities. In our framework, the audio clips are represented as log-mel-scale spectrograms of size 96×64, obtained by a short-time Fourier transform. The VGGish network converts the audio into 128-dimensional feature vectors with semantic information, where high-level feature vectors have more expressive power.

### 3.3. Multi-Modal Feature Fusion

Most dense video caption tasks rely on visual features, and a few integrate multi-modal features by concatenating or sharing the weights of different features rather than fully fusing them. As a result, multi-modal features are not fully functional in nature. To address this problem, we propose a multi-modal feature fusion approach that includes a multi-modal encoder, proposal heads, and confidence module. The multi-modal encoder stacks visual ($V_{n}$) and audio ($A_{n}$) features into *N* multi-modal encoder blocks to enable full fusion of the two features. Each multi-modal encoder block consists of self-attentive, multi-head attention, and fully connected layers. The process is described as follows. First, the self-attentive layer of the Transformer processes variable-length information sequences and dynamically generates different weights for the extracted visual and auditory features.

(1) $$\mathrm{self}\mathrm{Attention}\left(Q,K,V\right)=\mathrm{Softmax}\left(\frac{QK^{T}}{\sqrt{d}}\right)V,$$

where *Q*, *K*, and *V* denote query, key, and value, respectively. The $\sqrt{d}$ as a training parameter controls the gradient of Softmax with the purpose of enhancing the attention weights and distinguishing the differences between these features.

(2) $$\begin{array}{cc} V_{n}^{\mathrm{self}} & =\mathrm{self}\mathrm{Attention}\left(V_{n-1}^{\mathrm{fc}},V_{n-1}^{\mathrm{fc}},V_{n-1}^{\mathrm{fc}}\right), \end{array}$$

(3) $$\begin{array}{cc} A_{n}^{\mathrm{self}} & =\mathrm{self}\mathrm{Attention}\left(A_{n-1}^{\mathrm{fc}},A_{n-1}^{\mathrm{fc}},A_{n-1}^{\mathrm{fc}}\right). \end{array}$$

Then, multiple queries (*Q*) of multi-head attention are obtained to compute and produce a score for determination. These heads are concatenated as an output feature after the multi-head attentions are determined.

(4) $${\mathrm{head}}_{h}\left(Q,K,V\right)=\mathrm{self}\mathrm{Attention}\left(QW_{h}^{Q},KW_{h}^{K},VW_{h}^{V}\right),\;h\in \left[1,H\right].$$

All ${\mathrm{head}}_{h}$ will be concatenated and input to the multi-head attention:

(5) $$\mathrm{MultiHeadAttention}\left(Q,K,V\right)=\left[{\mathrm{head}}_{1}\left(Q,K,V\right),\ldots ,{\mathrm{head}}_{H}\left(Q,K,V\right)\right]W^{\mathrm{out}}.$$

Note that there are two different dimensions of multi-head attention weights produced by the two modalities, so concatenation processing is required to fuse them together.

(6) $$\begin{array}{cc} V_{n}^{A} & =\mathrm{MultiHeadAttention}\left(V_{n}^{\mathrm{self}},A_{n}^{\mathrm{self}},A_{n}^{\mathrm{self}}\right), \end{array}$$

(7) $$\begin{array}{cc} A_{n}^{V} & =\mathrm{MultiHeadAttention}\left(A_{n}^{\mathrm{self}},V_{n}^{\mathrm{self}},V_{n}^{\mathrm{self}}\right). \end{array}$$

At this point, the module will produce two fully merged new feature sequences: the visual feature $V_{n}^{\mathrm{fc}}$ and audio feature $A_{n}^{\mathrm{fc}}$, which also contain the most interesting information for the visual and audio components in this part, respectively.

(8) $$\begin{array}{cc} V_{n}^{\mathrm{fc}} & =\mathrm{Fully}\mathrm{Connected}\left(V_{n}^{A}\right), \end{array}$$

(9) $$\begin{array}{cc} A_{n}^{\mathrm{fc}} & =\mathrm{Fully}\mathrm{Connected}\left(A_{n}^{V}\right). \end{array}$$

Once the visual and audio features have been corrected, they are passed to the proposal heads to predict a set of proposal tags to initialize the video. This process can help select features that match the video content and improve the accuracy of the captioning module. However, due to differences in sequence lengths between the video and audio modalities, the feature sequences of the two modalities cannot match every proposal in the video at every time step. To alleviate this problem, a Common Pool is introduced to store the video or audio modality proposal corresponding to each time stamp. This allows filtering out noise and redundant information based on confidence scores.

**Figure 3:**
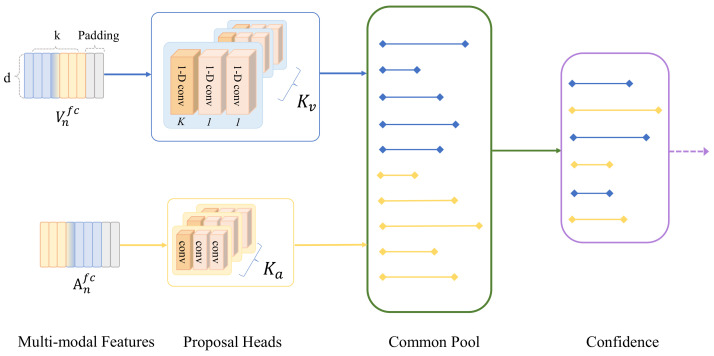
After the multi-modal encoder, the output features are marked in the proposal heads. The Common Pool is used to store the proposals predicted for each mode at each time step, and extract more important proposals by confidence.

As shown in Figure 3, there are two proposal heads $K_{v}$ (video) and $K_{a}$ (audio) as input features, which are passed to the proposal model in parallel. Each proposal head is a fully-CNN model consisting of three convolutional layers with different kernel sizes, and their paddings are used to unify the sequence length between each layer. The kernel size of the first convolutional layer is configured as k×k, which is used to scale-down the input size while extracting the most important features. Next, the kernel size of the other two convolutional layers is set to 1, with the goal of performing trainable weight batching for tensor learning. Each layer is separated by an activation function ReLU, and a Dropout layer is connected to the end in order to avoid overfitting.

The purpose of the Common Pool is to store proposals from different modalities, which are feature vectors extracted from video or audio modalities that reflect the content and semantics at each timestamp. The Common Pool puts these feature vectors into a shared space, allowing for comparison and communication between different modalities. Then, by using contrastive learning methods, the Common Pool can learn a unified representation that enables alignment and interaction between cross-modal proposals, thus providing a basis for subsequent processing and fusion.

We aim to select the most accurate proposals from the Common Pool based on their confidence score. We use the top-100 accuracy as a metric to evaluate the quality of the proposals and filter out the ones that are redundant or irrelevant. To further refine the proposals, we apply the *K*-means clustering algorithm with a Euclidean distance metric to estimate the optimal size of the kernel for each proposal. The kernel size is determined by predicting the threshold and receptive range that correspond to various high probability events in the feature space. We then scale the feature time span according to the clustering centroids and use them to obtain the values in grid cell coordinates. This way, we can generate more precise and compact proposals for different modalities. The details are as follows,

(10) $$c_{p}=p_{i}+\theta \left(l_{c}\right),$$

where $p_{i}=\left(\mathrm{start},\mathrm{end},\mathrm{confidence}\right)$, $i\in \left[1,100\right]$, and $l_{c}$ is the length. This $\theta \left(l_{c}\right)$ represents the $c_{p}$ (centre) position relative to position *p* in the sequences. The Sigmoid function θ ensures that the range must be $\left[0,1\right]$.

(11) $$\begin{array}{cc} \mathrm{start} & =c_{p}-\frac{l_{c}}{2}, \end{array}$$

(12) $$\begin{array}{cc} \mathrm{end} & =c_{p}+\frac{l_{c}}{2}, \end{array}$$

(13) $$\begin{array}{cc} \;\mathrm{confidence} & =\theta \left(l_{o}\right), \end{array}$$

where the proposal $p_{i}$ can be judged by the time bounds of “begin” and “end”, as well as the confidence scores, as in (13). Finally, the accuracy of the top 100 is obtained and the encoder is used to process the pruned features. Overall, the feature vectors with rich event relationships can be obtained after completing the multi-modal feature fusion module, which is important for generating comprehensive and logical text descriptions in this work.

### 3.4. Caption Generation

The decoder part of the model takes as input the feature vector output by the multi-modal feature fusion module and the previously generated word embedding representation. These inputs are then passed to the decoder layer for processing. We recommend using the LSTM as the RNN decoder part because it can effectively alleviate gradient explosion and remember long sequences. The LSTM iterates the next predicted word and generates the description text. Compared with the Transformer, LSTM also has good performance and can greatly reduce the model training time. Moreover, the output features of the last layer of the decoder can be used in the generator to predict the next caption word. The LSTM-based headline generation module is implemented by the following,

(14) $$I_{t}=\left\{\sum_{i=1}^{N}\exp\left(\theta \left(l_{c}\right)\right)\left(V_{n}^{\mathrm{fc}},A_{n}^{\mathrm{fc}}\right),W_{c}^{t}\right\},$$

where $I_{t}$ represents the initial state of the current caption generation module, $V_{n}^{\mathrm{fc}}$ and $A_{n}^{\mathrm{fc}}$ represent the video feature, including part of the audio and video feature, respectively. The $\exp\left(\cdot \right)$ function is used to determine the weight of the input feature, and $W_{c}^{t}$ denotes the subtitle word embedding feature. An input rule of LSTM is expressed in (15),

(15) $$h_{t}=\mathrm{LSTM}\left(y_{t-1},h_{t-1},I_{t-1}\right),$$

which is used to produce the current hidden state $h_{t}$. It receives the caption word embedding feature *y*, and $h_{t-1}$ and $l_{t-1}$ denote the two inter-hidden states that must be considered in each recurrent. The Softmax layer is used to compute the probability distribution *p* for the word prediction of the video caption,

(16) $$p\left(y_{t}|y_{t-1}\right)=\mathrm{Softmax}\left(y_{t-1},h_{t},I_{t}\right).$$

According to the predicted index of the vocabulary, the determined word can be continuously embedded and pass the net recurrent for the next word prediction until the end-of-sentence (<EOS>) symbol is received. Finally, the predictions are measured against the ground truth results, the difference is calculated using the cross-entropy function $L_{\mathrm{CE}}\left(\mu \right)$, and the gradient is contributed to the training energy. Given *g*, the reference caption in the dataset is the ground truth, μ is the training parameter in the proposed model, and a complete cross-entropy function is thus expressed as follows,

(17) $$L_{\mathrm{CE}}\left(\mu \right)=-\sum_{t=1}^{T}\log\left(p_{\mu }\left(g_{t}|g_{t-1}\right)\right).$$

## 4. Experiment

To evaluate the effectiveness of the proposed model, we performed experiments on the public ActivityNet Captions dataset and compared it with state-of-the-art methods.

### 4.1. Dataset and Data Pre-Processing

In our experiments, MSVD [61], ActivityNet Captions [31], and YouCook2 [62] are commonly used public datasets in the field of video caption. ActivityNet is mainly designed for dense video captioning tasks and covers a wide range of domains, which is exactly what our method requires. The ActivityNet Captions dataset contains 20,000 video clips with an average duration of 2 min. Each video is labeled with the events it contains, and the start and end times of each event are clearly marked, along with a manually created description of the event content. Note that some videos have been removed or altered by the original author and are no longer directly downloadable from the online resource. Following the approach used by most scholars, there are 10,024 videos in the training set, 4926 in the validation set, and 5044 in the test set. However, the labels in the test set are not yet publicly available, so we use the validation set for experiments and comparisons.

For the training preprocessing, the truecase, tokenization, and cleaning symbols must be completed, and the start mark <BOS> and end mark <EOS> must be inserted at the beginning and end of a sentence, respectively. Due to the limited size of the vocabulary and the misleading description of low-frequency word pairs, words with a frequency of less than 5 in the text are uniformly replaced by <UNK>, whose semantics are discarded and considered to be out of the vocabulary. To prevent no input from the decoder at the beginning, the <BOS> is also inserted as the first token, and the caption will be generated verbatim until a unique end token <EOS> is also inserted.

### 4.2. Implementation

The environment we set up was a Ubuntu 20.04 system, and we made use of PyTorch [63] as the neural network engine for our implementation. These experiments were trained on NVIDIA GeForce RTX 3070Ti GPUs with 8.0 GB of device memory. The I3D network had been pre-trained on the Kinetics dataset used in the visual feature extraction stage. The input consisted of RGB features extracted at 25.0 fps and 64 optical flow features of size $224^{2}$. The dimension of the output features was 1024. Additionally, audio features were extracted by VGGish pre-trained on AudioSet, where the pre-classification layer embedded 128 dimensions for each feature, and configured the batch size as 32. In addition, the learning rate was initialized to $10^{-4}$, and Adam [64] was used as the optimizer.

For the multi-modal feature fusion module, we took features of different sizes and mapped them to an inner space with a 1024-dimensional vector. Then, we used two different sizes of features as 128 features for visual and 48 features for audio, and stacked N=2 and H=4 in multi-head attention. In Section 3.3, we configured the proposal header with $K_{v}=K_{a}=10$, while for the kernel *K*, we used a different size. The size of the visual modality was determined after calculating the *K* mean. Next, the LSTM was connected to the proposed heads that received the hidden state and performed the decoding. These results were passed to Softmax to compute the probability of the next word determination. In practice, the word embedding size was 468 and the learning rate was 5 × $10^{-5}$. The localization and target loss factor was 1.0. To maintain a balance between the two modalities, we set the size of the two hidden layers of the proposal header to 512, so the input size of the fully connected layer was also 512.

### 4.3. Results and Analysis

To demonstrate the performance of the proposed framework, we performed validation on the ActivityNet Captions dataset and compared it with various state-of-the-art methods. We provide ablation studies to validate the impacts of the individual modules in our framework on the experimental results. We also report the results of the qualitative analysis, which highlights the superiority of our proposed model.

#### 4.3.1. Comparison to the State-of-the-Art

We compared the proposed model with various state-of-the-art methods on the dense video caption tasks, including: EEDVC [46], DCE [31], MFT [32], WLT [49], SDVC [33], EHVC [25], MDVC [55], BMT [54], PDVC [47], and EMVC [56]. The results of the comparison are shown in Table 2.

Among them, B@N represents the evaluation metric BLEU [65], which compares the degree of overlapping of *N*-grams between translated results of predicted and reference. It is widely used to evaluate the level of text expression in neural machine translation (NMT). Furthermore, METEOR [36] considers the recall rate and accuracy rate based on BLEU, and uses the F-Value as the final evaluation metric. Moreover, CIDEr [66] mainly calculates the similarity between predicted and reference sentences, whose principle meets the evaluation requirements in the field of image and video caption. Higher values of these evaluation metrics indicate better performance of the generated text description.

All experimental results are shown in Table 2, and the proposed work achieved better results than the others. Note that the SDVC model incorporates reinforcement learning in the training process, so the score of BLEU-1 was higher than our method. It is worth noting that the proposed model outperformed both BMT and EMVC in the scores of each item, and their approaches were also based on audiovisual feature fusion for intensive video caption generation. They differ from our model in that both models use the Bi-Transformer in both the encoder and decoder, and we use the Transformer framework only in the encoder; we instead use the LSTM as the decoder for generating descriptive text. In addition, some missing parts of the ActivityNet Captions dataset did not provide complete metrics for BMT and EMVC. Even so, this still proved that our method not only required less training time, but also performed competitively.

#### 4.3.2. Ablation Study

We conducted a large number of comparative experiments to verify the impact of different components of the proposed model on the output results, including multi-modal feature extraction, a Transformer for multi-modal feature fusion, and an LSTM for caption generation.

Table 3 indicates the impact of multi-modal features on the generated captions. The results using fused audio and visual features always outperform the best under different evaluation metrics. In all three cases, the results using pure audio modality features were the weakest, which means that the visual modality may contain more information about the video content. However, the difference between visual and audio remained fixed, but the fusion effect was good, indicating that audio played a certain role as additional feature information.

We also conducted an ablation study to verify the effectiveness of using Transformer’s multi-head attention to fully fuse multi-modal features in our model. We compared our method with a baseline method that used only multi-modal features for concatenation without attention. The results are shown in Table 4. We can see that our method outperforms the baseline method on all metrics, indicating that the proposed method can achieve a more complete and robust feature fusion by using Transformer. Moreover, we can observe that the proposed method can generate more comprehensive and accurate video captions than the baseline method, as it can capture more details and nuances from both visual and audio modalities.

We tested the effect of using LSTM, GRU, and the Transformer as decoders on the abstract representation of the video. As shown in Table 5, we observe that most of the methods that used Transformer as a decoder to generate text descriptions achieved slightly higher scores than the LSTM we employed. However, the difference was not significant, and our method still outperformed most of the Transformer-based methods on some metrics. Moreover, our method has the advantage of faster training and inference speed than other methods, as it requires less memory and computation. The time to generate text descriptions for each video was correspondingly shorter, which is desirable for practical applications. Therefore, we conclude that our approach is still competitive and efficient for dense video caption.

#### 4.3.3. Qualitative Analysis

**Figure 4:**
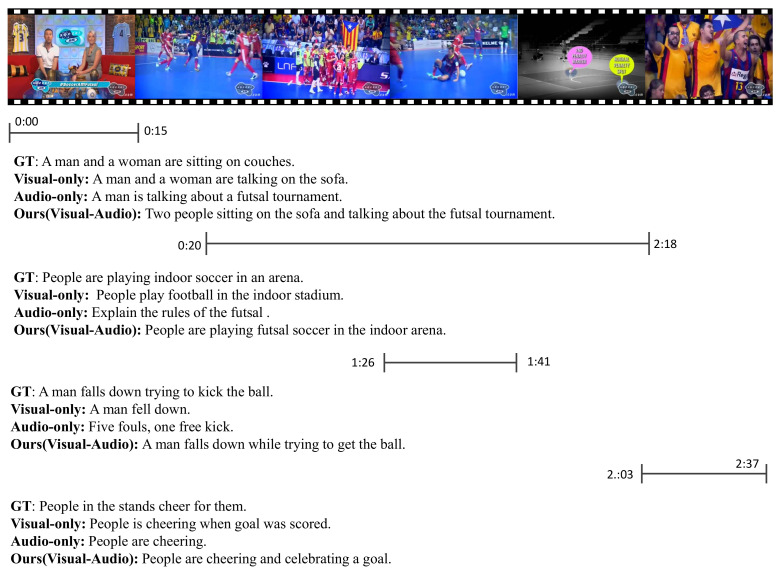
Results of a qualitative analysis of a video from the ActivityNet Caption validation dataset. The predicted results of the proposed model are compared to the visual-only model, the audio-only model, and the ground truth (GT) reference.

Furthermore, we demonstrated text description generation using the proposed multi-modal feature fusion method on the ActivityNet Captions dataset. We also compared the video captions generated with visual or audio-only features as input, as shown in Figure 4.

As the results show in Figure 4, both our method and the method using only audio features captured the keyword “futsal” at the beginning of the video time [0:00–0:15]. In contrast, the method using only visual features missed this keyword and only expressed the conversation between two people. It is impossible to obtain the specific content of the chat without the audio features as a complement, so the generated captions are not acceptable. Furthermore, at video time [1:26–1:41], there was a gap between the audio and visual features provided in this solution, so the auxiliary function of the audio features was not highlighted. The entire video lasted more than 2 min, and the captions generated by our proposed model were more detailed and accurate than the reference captions provided by the ground truth.

## 5. Conclusions

We propose a framework for enhancing the performance of dense video caption using a multi-modal feature fusion approach. The proposed framework employs I3D to extract visual features and VGGish to extract audio features from videos. The Transformer encodes the multi-modal features, and LSTM decodes them to generate descriptive text for the video. The overall framework is compact and efficient for training. To generate more accurate results, we fed the feature output from the encoder into the proposal head module. Moreover, to align the visual-audio features with different sequence lengths at each time step after the fusion, we use a Common Pool to predict and fuse each modality of every recurrent step. Furthermore, we use confidence scores to extract more consistent features for video content, which can improve the quality of the model-generated sentences.

Experiments show that the proposed multi-modal feature fusion model surpasses other approaches that use only visual or audio modality features. The model is also competitive with other dense video caption models. The text generated by the proposed model follows natural language rules, highlighting the importance of audio features for this task.

Our work still has shortcomings compared to the ground truth, and we cannot fully recognize the short-term killing parts. Future work could explore the addition of other modalities in video to enhance the expression of video content with more auxiliary information, increase the accuracy of computer understanding of videos, and improve the fine-grained caption generation.

## Figures and Tables

**Table 1:** Summary of selected video caption methods.

Method	CNN	RNN	Attention	Transformer	Visual	Audio	Others
S2VT [21], LSTM-E [37]	✓	✓			✓		
DCE [31], SST [35], STS [39], STaTS [40]	✓	✓	✓		✓		
AMT [42], SwinBERT [43], PDVC [47], TDPC [48]				✓	✓		
ETGS [51], VGA [57]	✓	✓	✓		✓	✓	
DVMF [50]	✓	✓	✓		✓	✓	✓
MDVC [55], BMT [54], EMVC [56], FiI [58]				✓	✓	✓	

**Table 2:** Comparison of the performance of our proposed method with state-of-the-art methods on the ActivityNet Captions dataset. The **bold** fonts indicate the best results.

Models	B@1	B@2	B@3	B@4	METEOR	CIDEr
EEDVC [46]	9.96	4.81	2.91	1.44	6.91	9.25
DCE [31]	10.81	4.57	1.90	0.71	5.69	12.43
MFT [32]	13.31	6.13	2.84	1.24	7.08	21.00
WLT [49]	10.00	4.20	1.85	0.90	4.93	13.79
SDVC [33]	**17.92**	7.99	2.94	0.93	8.82	-
EHVC [25]	-	-	-	1.29	7.19	14.71
MDVC [55]	12.59	5.76	2.53	1.01	7.46	7.38
BMT [54]	13.75	7.21	3.84	1.88	8.44	11.35
PDVC [47]	-	-	-	**1.96**	8.08	28.59
EMVC [56]	14.65	7.10	3.23	1.39	9.64	13.29
**Proposed**	16.77	**8.15**	**4.03**	1.91	**10.24**	**32.82**

**Table 3:** The impact of proposed multi-modal features on generated captions. The **bold** fonts indicate the best results.

Modality	B@1	B@2	B@3	B@4	METEOR	CIDEr
Visual-only	13.71	7.08	2.58	1.15	6.98	18.36
Audio-only	12.14	6.27	2.64	1.03	5.82	15.74
**Proposed**	**16.77**	**8.15**	**4.03**	**1.91**	**10.24**	**32.82**

**Table 4:** Comparing the impact of different fusion methods on proposed multi-modal features. The **bold** fonts indicate the best results.

Method	B@1	B@2	B@3	B@4	METEOR	CIDEr
Concatenate	14.84	5.19	3.61	1.66	7.53	25.47
**Proposed**	**16.77**	**8.15**	**4.03**	**1.91**	**10.24**	**32.82**

**Table 5:** The effect of different decoder on dense video caption. The **bold** fonts indicate the best results.

Decoder	B@1	B@2	B@3	B@4	METEOR	CIDEr
Transformer	**18.14**	**8.29**	**4.12**	1.87	**10.31**	**33.46**
GRU	15.57	6.56	3.81	1.64	8.73	28.95
**LSTM**	16.77	8.15	4.03	**1.91**	10.24	32.82

## Data Availability

Not applicable.

## References

[1] Jain A.K., Sahoo S.R., Kaubiyal J. (2021). Online social networks security and privacy: Comprehensive review and analysis. Complex Intell. Syst..

[2] Wu Y., Sheng H., Zhang Y., Wang S., Xiong Z., Ke W. (2022). Hybrid motion model for multiple object tracking in mobile devices. IEEE Internet Things J..

[3] Sheng H., Lv K., Liu Y., Ke W., Lyu W., Xiong Z., Li W. (2020). Combining pose invariant and discriminative features for vehicle reidentification. IEEE Internet Things J..

[4] Shapiro L.G. (2018). Computer vision: The last 50 years. Int. J. Parallel Emerg. Distrib. Syst..

[5] Wang S., Sheng H., Yang D., Zhang Y., Wu Y., Wang S. (2022). Extendable multiple nodes recurrent tracking framework with RTU++. IEEE Trans. Image Process..

[6] Sheng H., Wang S., Zhang Y., Yu D., Cheng X., Lyu W., Xiong Z. (2020). Near-online tracking with co-occurrence constraints in blockchain-based edge computing. IEEE Internet Things J..

[7] Zhang W., Ke W., Yang D., Sheng H., Xiong Z. (2023). Light field super-resolution using complementary-view feature attention. Comput. Vis. Media.

[8] Chowdhary K.R. (2020). Natural Language Processing. Fundamentals of Artificial Intelligence.

[9] Chan K.H., Im S.K., Pau G. Applying and Optimizing NLP Model with CARU. Proceedings of the 2022 8th International Conference on Advanced Computing and Communication Systems (ICACCS).

[10] Ke W., Chan K.H. (2021). A Multilayer CARU Framework to Obtain Probability Distribution for Paragraph-Based Sentiment Analysis. Appl. Sci..

[11] Sheng H., Zheng Y., Ke W., Yu D., Cheng X., Lyu W., Xiong Z. (2020). Mining hard samples globally and efficiently for person reidentification. IEEE Internet Things J..

[12] LeCun Y., Bengio Y., Hinton G. (2015). Deep learning. Nature.

[13] Sawarn A., Srivastava S., Gupta M., Srivastava S. (2021). BeamAtt: Generating Medical Diagnosis from Chest X-rays Using Sampling-Based Intelligence. EAI/Springer Innovations in Communication and Computing.

[14] Pan Y., Wang L., Duan S., Gan X., Hong L. (2021). Chinese image caption of Inceptionv4 and double-layer GRUs based on attention mechanism. J. Phys. Conf. Ser..

[15] Wang S., Sheng H., Zhang Y., Wu Y., Xiong Z. A general recurrent tracking framework without real data. Proceedings of the 2021 IEEE/CVF International Conference on Computer Vision (ICCV).

[16] Zhang S., Lin Y., Sheng H. Residual networks for light field image super-resolution. Proceedings of the 2019 IEEE/CVF Conference on Computer Vision and Pattern Recognition (CVPR).

[17] Jiao Y., Chen S., Jie Z., Chen J., Ma L., Jiang Y.G. (2022). More: Multi-order relation mining for dense captioning in 3d scenes. Proceedings of the Computer Vision—ECCV.

[18] Venugopalan S., Xu H., Donahue J., Rohrbach M., Mooney R., Saenko K. (2015). Translating Videos to Natural Language Using Deep Recurrent Neural Networks. Proceedings of the 2015 Conference of the North American Chapter of the Association for Computational Linguistics: Human Language Technologies.

[19] Huang X., Ke W., Sheng H. (2022). Enhancing Efficiency and Quality of Image Caption Generation with CARU. Wireless Algorithms, Systems, and Applications.

[20] Aafaq N., Mian A.S., Akhtar N., Liu W., Shah M. (2022). Dense video captioning with early linguistic information fusion. IEEE Trans. Multimed..

[21] Venugopalan S., Rohrbach M., Donahue J., Mooney R., Darrell T., Saenko K. Sequence to Sequence—Video to Text. Proceedings of the 2015 IEEE International Conference on Computer Vision (ICCV).

[22] Simonyan K., Zisserman A. (2014). Very Deep Convolutional Networks for Large-Scale Image Recognition. arXiv.

[23] Hochreiter S., Schmidhuber J. (1997). Long Short-Term Memory. Neural Comput..

[24] Tran D., Bourdev L., Fergus R., Torresani L., Paluri M. Learning Spatiotemporal Features with 3D Convolutional Networks. Proceedings of the 2015 IEEE International Conference on Computer Vision (ICCV).

[25] Wang T., Zheng H., Yu M., Tian Q., Hu H. (2021). Event-Centric Hierarchical Representation for Dense Video Captioning. IEEE Trans. Circuits Syst. Video Technol..

[26] Hara K., Kataoka H., Satoh Y. Can Spatiotemporal 3D CNNs Retrace the History of 2D CNNs and ImageNet? In Proceedings of the 2018 IEEE/CVF Conference on Computer Vision and Pattern Recognition, Salt Lake City, UT, USA, 18–22 June 2018.

[27] Zhang Y., Sheng H., Wu Y., Wang S., Lyu W., Ke W., Xiong Z. (2020). Long-term tracking with deep tracklet association. IEEE Trans. Image Process..

[28] Wang S., Yang D., Wu Y., Liu Y., Sheng H. (2022). Tracking Game: Self-adaptative Agent based Multi-object Tracking. Proceedings of the Proceedings of the 30th ACM International Conference on Multimedia.

[29] Carreira J., Zisserman A. Quo Vadis, Action Recognition? A New Model and the Kinetics Dataset. Proceedings of the 2017 IEEE Conference on Computer Vision and Pattern Recognition (CVPR).

[30] Qiu Z., Yao T., Mei T. Learning Spatio-Temporal Representation with Pseudo-3D Residual Networks. Proceedings of the 2017 IEEE International Conference on Computer Vision (ICCV).

[31] Krishna R., Hata K., Ren F., Fei-Fei L., Niebles J.C. Dense-Captioning Events in Videos. Proceedings of the 2017 IEEE International Conference on Computer Vision (ICCV).

[32] Xiong Y., Dai B., Lin D. (2018). Move Forward and Tell: A Progressive Generator of Video Descriptions. Proceedings of the European Conference on Computer Vision (ECCV) 2018.

[33] Mun J., Yang L., Ren Z., Xu N., Han B. Streamlined Dense Video Captioning. Proceedings of the 2019 IEEE/CVF Conference on Computer Vision and Pattern Recognition (CVPR).

[34] Yu H., Wang J., Huang Z., Yang Y., Xu W. Video Paragraph Captioning Using Hierarchical Recurrent Neural Networks. Proceedings of the 2016 IEEE Conference on Computer Vision and Pattern Recognition (CVPR).

[35] Buch S., Escorcia V., Shen C., Ghanem B., Niebles J.C. SST: Single-Stream Temporal Action Proposals. Proceedings of the 2017 IEEE Conference on Computer Vision and Pattern Recognition (CVPR).

[36] Banerjee S., Lavie A. (2005). METEOR: An Automatic Metric for MT Evaluation with Improved Correlation with Human Judgments. Proceedings of the ACL Workshop on Intrinsic and Extrinsic Evaluation Measures for Machine Translation and/or Summarization.

[37] Pan Y., Mei T., Yao T., Li H., Rui Y. Jointly Modeling Embedding and Translation to Bridge Video and Language. Proceedings of the 2016 IEEE Conference on Computer Vision and Pattern Recognition (CVPR).

[38] Baraldi L., Grana C., Cucchiara R. Hierarchical Boundary-Aware Neural Encoder for Video Captioning. Proceedings of the 2017 IEEE Conference on Computer Vision and Pattern Recognition (CVPR).

[39] Yao L., Torabi A., Cho K., Ballas N., Pal C., Larochelle H., Courville A. (2015). Video description generation incorporating spatio-temporal features and a soft-attention mechanism. arXiv.

[40] Cherian A., Wang J., Hori C., Marks T.K. Spatio-Temporal Ranked-Attention Networks for Video Captioning. Proceedings of the 2020 IEEE Winter Conference on Applications of Computer Vision (WACV).

[41] Gabeur V., Sun C., Alahari K., Schmid C. (2020). Multi-modal Transformer for Video Retrieval. Computer Vision—ECCV 2020.

[42] Yu Z., Han N. (2021). Accelerated masked transformer for dense video captioning. Neurocomputing.

[43] Lin K., Li L., Lin C.C., Ahmed F., Gan Z., Liu Z., Lu Y., Wang L. (2021). SwinBERT: End-to-End Transformers with Sparse Attention for Video Captioning. arXiv.

[44] Zhang S., Sheng H., Yang D., Zhang J., Xiong Z. (2017). Micro-lens-based matching for scene recovery in lenslet cameras. IEEE Trans. Image Process..

[45] Zhong R., Zhang Q., Zuo M. (2023). Enhanced visual multi-modal fusion framework for dense video captioning. Res. Sq..

[46] Zhou L., Zhou Y., Corso J.J., Socher R., Xiong C. End-to-End Dense Video Captioning with Masked Transformer. Proceedings of the 2018 IEEE/CVF Conference on Computer Vision and Pattern Recognition.

[47] Wang T., Zhang R., Lu Z., Zheng F., Cheng R., Luo P. End-to-End Dense Video Captioning with Parallel Decoding. Proceedings of the 2021 IEEE/CVF International Conference on Computer Vision (ICCV).

[48] Song Y., Chen S., Jin Q. Towards diverse paragraph captioning for untrimmed videos. Proceedings of the 2021 IEEE/CVF Conference on Computer Vision and Pattern Recognition.

[49] Rahman T., Xu B., Sigal L. Watch, Listen and Tell: Multi-Modal Weakly Supervised Dense Event Captioning. Proceedings of the 2019 IEEE/CVF International Conference on Computer Vision (ICCV).

[50] Jin Q., Chen J., Chen S., Xiong Y., Hauptmann A. (2016). Describing videos using multi-modal fusion. Proceedings of the 24th ACM International Conference on Multimedia.

[51] Chen S., Jin Q., Chen J., Hauptmann A.G. (2019). Generating Video Descriptions with Latent Topic Guidance. IEEE Trans. Multimed..

[52] Martinez J., Perez H., Escamilla E., Suzuki M.M. Speaker recognition using Mel frequency Cepstral Coefficients (MFCC) and Vector quantization (VQ) techniques. Proceedings of the CONIELECOMP 2012, 22nd International Conference on Electrical Communications and Computers.

[53] Hershey S., Chaudhuri S., Ellis D.P.W., Gemmeke J.F., Jansen A., Moore R.C., Plakal M., Platt D., Saurous R.A., Seybold B. CNN architectures for large-scale audio classification. Proceedings of the 2017 IEEE International Conference on Acoustics, Speech and Signal Processing (ICASSP).

[54] Iashin V., Rahtu E. (2020). A better use of audio-visual cues: Dense video captioning with bi-modal transformer. arXiv.

[55] Iashin V., Rahtu E. Multi-modal dense video captioning. Proceedings of the 2020 IEEE/CVF Conference on Computer Vision and Pattern Recognition Workshops (CVPRW).

[56] Chang Z., Zhao D., Chen H., Li J., Liu P. (2022). Event-centric multi-modal fusion method for dense video captioning. Neural Netw..

[57] Hao W., Zhang Z., Guan H. Integrating both visual and audio cues for enhanced video caption. Proceedings of the Thirty-Second AAAI Conference on Artificial Intelligence.

[58] Park J.S., Darrell T., Rohrbach A. (2020). Identity-Aware Multi-sentence Video Description. Computer Vision—ECCV 2020.

[59] Carreira J., Noland E., Hillier C., Zisserman A. (2019). A Short Note on the Kinetics-700 Human Action Dataset. arXiv.

[60] Gemmeke J.F., Ellis D.P.W., Freedman D., Jansen A., Lawrence W., Moore R.C., Plakal M., Ritter M. Audio Set: An ontology and human-labeled dataset for audio events. Proceedings of the 2017 IEEE International Conference on Acoustics, Speech and Signal Processing (ICASSP).

[61] Chen D., Dolan W. Collecting Highly Parallel Data for Paraphrase Evaluation. Proceedings of the 49th Annual Meeting of the Association for Computational Linguistics: Human Language Technologies.

[62] Zhou L., Xu C., Corso J. Towards Automatic Learning of Procedures from Web Instructional Videos. Proceedings of the Thirty-Second AAAI Conference on Artificial Intelligence.

[63] Paszke A., Gross S., Massa F., Lerer A., Bradbury J., Chanan G., Killeen T., Lin Z., Gimelshein N., Antiga L., Wallach H., Larochelle H., Beygelzimer A., d’Alché Buc F., Fox E., Garnett R. (2019). PyTorch: An Imperative Style, High-Performance Deep Learning Library. Advances in Neural Information Processing Systems 32.

[64] Kingma D.P., Ba J. (2014). Adam: A Method for Stochastic Optimization. arXiv.

[65] Papineni K., Roukos S., Ward T., Zhu W.J. (2002). BLEU: A method for automatic evaluation of machine translation. Proceedings of the 40th Annual Meeting on Association for Computational Linguistics—ACL’02.

[66] Vedantam R., Zitnick C.L., Parikh D. CIDEr: Consensus-based image description evaluation. Proceedings of the 2015 IEEE Conference on Computer Vision and Pattern Recognition (CVPR).

